# Biomedical semantics in the Semantic Web

**DOI:** 10.1186/2041-1480-2-S1-S1

**Published:** 2011-03-07

**Authors:** Andrea Splendiani, Albert Burger, Adrian Paschke, Paolo Romano, M Scott Marshall

**Affiliations:** 1 Rothamsted Research, AL5 J2Q, Harpenden, UK; 2 Heriot-Watt University and MRC Human Genetics Unit, Edinburgh, UK; 3 Freie Universitaet Berlin, Berlin, Germany; 4 National Cancer Research Institute of Genoa, I-16132, Italy; 5 Leiden University Medical Center / University of Amsterdam, The Netherlands

## Abstract

The Semantic Web offers an ideal platform for representing and linking biomedical information, which is a prerequisite for the development and application of analytical tools to address problems in data-intensive areas such as systems biology and translational medicine. As for any new paradigm, the adoption of the Semantic Web offers opportunities and poses questions and challenges to the life sciences scientific community: which technologies in the Semantic Web stack will be more beneficial for the life sciences? Is biomedical information too complex to benefit from simple interlinked representations? What are the implications of adopting a new paradigm for knowledge representation? What are the incentives for the adoption of the Semantic Web, and who are the facilitators? Is there going to be a Semantic Web revolution in the life sciences?

We report here a few reflections on these questions, following discussions at the SWAT4LS (Semantic Web Applications and Tools for Life Sciences) workshop series, of which this Journal of Biomedical Semantics special issue presents selected papers from the 2009 edition, held in Amsterdam on November 20^th^.

## Introduction

The increasing amounts of data being gathered on biological systems and the convergence of different disciplines are leading to entirely new areas of research, from systems biology to translational and personalized medicine. These, in turn, promise to have a significant impact on our society. This promise relies on the multi-disciplinary integration and analysis of data. However, biomedical information is challenging: it is heterogeneous, fragmented and characterized by a complex semantics [1]. The many properties and attributes that characterize biological phenomena give biomedical data its multi-dimensional nature. Additional complexity arises through layers of systems and entities that interact at multiple levels of granularity, from the molecular level to the macro level of the organism and environment.

The Semantic Web is a set of standards and technologies which provides tools to address such challenges, by enabling an explicit characterization of the semantics of information, by which heterogeneous and distributed information can be linked. For example, the Semantic Web can easily represent the network model of molecular and system interactions that researchers refer to in pathways. Such pathways may be represented as paths through a graph or (semantic) web of connections between information resources representing the entities which participate in such pathways. The first mention of the potential of these technologies to address the increasing complexity of life sciences information dates back to 2001 [2], just after the completion of the Human Genome Project. Since then, a number of papers have discussed the potential impact of these technologies [3-6], have reported success stories [7], have argued over their limits [8,9] or have presented reviews on state of the art of their application [10-12].

The Semantic Web is now becoming mature. Some of its standards, such as RDF (Resource Description Framework) and SPARQL (an RDF Query Language), are supported by several tools which are produced by an emerging ecosystem of enterprises. These tools and standards are being adopted both by governments and by major information industry players [13-17]. Outside the life sciences domain, recent developments in the Web community demonstrate that key players are adopting the Semantic Web standards. For example, Twitter, Facebook, Drupal, and Google have each separately announced various ways to produce and consume RDF using their APIs. Twitter announced a new feature called Annotations that enables users to add rich metadata to their tweets. Facebook has adopted RDF-based Open Graph Protocol to link sites to Facebook. Drupal 7 includes RDF mappings for the most common content types and allows Drupal-based distributions to predefine their data structure using RDF, with SPARQL integration being promised in the near future. Google has started to recommend the GoodRelations ontology for RDFa (RDF-in- attributes) markup of Web pages for better e-commerce representation in search results lists. These small, but significant, developments show that important companies, projects, and institutions on the Web are seeing the value of the Semantic Web.

Many life sciences researchers have been early adopters of Semantic Web technologies. SWAT4LS (Semantic Web Applications and Tools for Life Sciences) is a workshop series whose main objective is to foster a critical discussion about the possibilities, limitations, and adoption of the Semantic Web in the life sciences and in biomedical research.

In addition to previous editions of this workshop, SWAT4LS 2008 [18] and SWAT4LS 2009 [19], the Semantic Web has been the focus of both the 2010 edition of the Bio-Ontology workshop [20] and the Biohackathon [21]. Life sciences also played a prominent role in many sessions of the 2010 Semantic Technology Conference [22]. The Concept Web Alliance [23], whose initial focus since inception in 2009 has been in the life sciences, has also been founded on the Semantic Web principles and technologies. The importance of the Semantic Web has also been recognized by research sponsors: the National Center for Biomedical Ontologies (NCBO) at Stanford, whose BioPortal offers a set of Biomedical Semantic Web services, is receiving another round of funding as one of the NIH National Centers for Biomedical Computing in the U.S.

**Figure 1:**
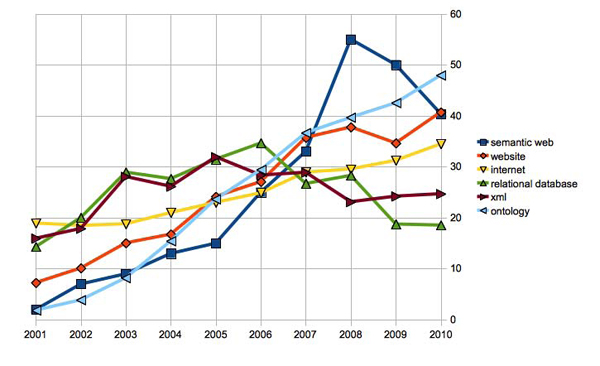
**Number of papers in Pubmed which contain the keyword “Semantic Web”, and related keywords, published during the years 2001-2010.** Numbers of papers in Pubmed from 2001 to 2010 which contain in the title or abstract the keywords “Semantic Web”, “Website”, “Internet”, “Relational database”, “XML”, “Ontology”. The number of papers for 2010 is linearly extrapolated from the total number of papers published until November 2010. The numbers reported are first normalized by the total number of papers published in Pubmed per year (multiplied by a factor of 10^5^ for readability). They are then divided by the following ratio: (total numbers of papers published in category X between 2001-2010)/(total numbers of papers published in category “Semantic Web” between 2001-2010)

It is perhaps surprising, then, that the number of papers which explicitly mention the term “Semantic Web” in Pubmed has shown a decline in the last two years. Figure 1 shows the trend over the period from 2001 to 2010, in comparison with a few other keywords representing related technologies. While the terms “website”, “internet” and “ontology” are appearing more and more in abstracts, the term “Semantic Web” which ought to encompass them, is not. Could this be due to a general preference for the terms that refer to particular aspects of the Semantic Web such as “ontology” and “linked data"? Clearly, Figure 1 is only a weak and imprecise indication of the impact of a technology, as seen through the frequency of its related terms in literature. Nevertheless, this observation calls for some thoughts on why we do not see more of Semantic Web in life sciences.

## Which Semantic Web?

The two words that make up the term “Semantic Web” reveal its two cores: “the web” and “the semantics”. Indeed, one aim of the Semantic Web is to make data accessible on the Web (i.e. using web protocols such as HTTP), the most prominent example of this aim being perhaps represented by Linked Data [24]. In life sciences, only a few integration solutions focus on this “data access” aspect (we cite, as examples, Bio2RDF [25] and LODD[26]).

On the other hand, the desire to semantically characterize biological data is older than the web. Hence, the development of biomedical ontologies is already an active field, and is relatively mature in terms of use cases, infrastructures and methodologies [27,28]. These two cores of the Semantic Web are not clear-cut: ontologies are used to provide an interpretation of information presented in RDF [29]. OWL (the Ontology Web Language) is increasingly adopted for the definition of bio-ontologies [30,31]. However, there are still many life sciences information resources which expose ontologies and information by using custom technologies. The Semantic Web is not a monolithic set of technologies. “Which” Semantic Web we will see in the life sciences is still an open question: which balance of “web” and “semantics” will it involve?

## What does the data mean?

Once people start sharing data as RDF, they are confronted with a challenge that was never apparent before, often because it simply was not attempted: large-scale semantics-based data integration. The use of common identifiers to refer to the same data items can ease data integration tremendously, making an otherwise costly mapping and alignment process unnecessary. This is the intention of the Shared Names initiative, as well as of the PURL identifiers for terms from OBO ontologies and NCBO. However, even when basic entity types such as genes, proteins, and biological processes can be unambiguously named through either ontology or Shared Name identifiers, the process of alignment of data and semantics across domains and disciplines remains a challenge. It is this alignment or mapping process that is the most challenging to any data integration effort, and crucial to the success of efforts in research areas such as translational medicine and systems biology. When researchers attempt to align and integrate data from different sources, they are faced with the question of precisely what the data means - the semantics are not usually explicitly recorded during data collection and data management. What type of relation does a row in a spreadsheet from a microarray image analysis program represent? Does it simply represent the “gene expression” or a statistically interpreted (by an algorithm) measurement of fluorescence that is associated with the mRNA levels of the probe? If there are no previous examples of RDF representation for the data, the work of representation, choice of vocabularies, and alignment must occur at the time of data integration. In other words, data integration is challenging precisely because it requires semantic commitment and alignment, as well as the requisite understanding of the data.

## The "complexity ceiling"

Life sciences researchers have been early adopters of Semantic Web technologies. Many biomedical information resources are already available in RDF, some providing resolvable URIs (Uniform Resource Identifiers) and references to external resources. Within the datasets which are “integrated” using Linked Open Data [32], datasets related to life sciences account for less than the 10% of triples, but for more than the 50% of total outgoing links.

These results, however, may not be enough. Biomedical information is complex. It is related to a variety of disciplines which still lack a common language, and it cannot rely on a single domain of shared concepts as the basis of data integration: there is no common coordinate system such as that used in geographical map-based data mashups. Although chromosome position can work, as a common basis for comparison for many gene expression-related data [33], the genome cannot be used as a reference for all biological data integration. In another example, even a coordinate space for comparison of anatomical location remains a challenge: see efforts to normalize coordinate spaces across different sources of instrumentation for brain scans [34].

The diffusion of the Semantic Web in the life sciences could be bound by the normalization of knowledge representations and common identifiers, requiring an unprecedented degree of consensus across disciplines.

## The need for incentives and facilitators

Biomedical sciences have been a forerunner in the adoption of Open Access policies for publications and they went further: both major journals and grant agencies require the most relevant data to be released in public repositories by using standard formats. There is, however, no incentive for these repositories to open their information in a common, machine-processable format, such as RDF. Biomedical public repositories are like “open silos”: they store public data and provide APIs for programmatic access, but they lack a common representation. This is in contrast to the commitment to an open data infrastructure which has been recently adopted by such entities as the UK and US government for their own data: when stakeholders of such relevance start to publish information in open formats, they act as facilitators in the adoption of technologies and standards. The life sciences perhaps also need facilitators. Take for instance the requirement for stable, resolvable, URIs by major database providers, which is perhaps one of the most relevant steps to boost the development of Semantic Web technologies. There is a general lack of resources and motivation for the database providers to create and maintain such URIs, not to mention the need to coordinate with other consistent implementations. As a result, much of the initial experimental linked data has involved unresolvable URIs that employ ad hoc URI formats, e.g. “http://mysemweb.org/foo/name”. Shared Names [35], an initiative meant to supply permanent URIs that refer to information records in major databases with a uniform approach and federation of PURL (permanent URL) servers, is setting up common identifiers that will ease data integration and data sharing.

While the uptake of Semantic Web technologies in the life sciences   is gradually progressing, the endorsement by granting agencies, by   major journals as well as by main information providers would make it   more agile and coordinated.

## A change in paradigm

Occasionally, a lack of progress is lamented when discussing the Semantic Web. As a point of comparison, consider the evolution of relational databases (RDBs). Codd’ s original publication “A Relational Model of Data for Large Shared Data Banks” [36] was published in 1970 and the associated query language, SQL, has been standardized since 1986, with a variety of database implementations that now define data management practice for many companies and institutions. However, relational databases only became commonplace on industry servers in the 1990’ s, with confidence in reliability and experience in scalability growing into the following decade, when RDBs became a common fixture on personal computers. Clearly, it can take quite a few years for a new technology to be widely adopted and become stable. More recently, SGML was defined as a standard for mark-up of data in 1986. It led to the development of XML which, in turn, was standardized in 1998.

The establishment of new information representation paradigms therefore requires time. The Semantic Web is relatively young (RDF was first proposed in 1999, OWL in 2004 and OWL2 in 2009) and, in pursuing the development of the web into a distributed knowledge base, it proposes a major change of paradigm. The Semantic Web for instance moves away from the document and process metaphors, which are common in information systems: it is based on the properties of entities identified by URIs, not on the “containers” of such properties (i.e. documents that refer to the entities and their properties). The Semantic Web also makes explicit that the extent of information is unknown: when looking for information on the Web, it is even intuitively clear that what is found is only a subset of existing information. This is not the general assumption which is expected from an information resource.

## When is the revolution scheduled?

With 200 ontologies being served from NCBO’ s APIs and SPARQL endpoint, shouldn’ t there already be a Semantic Web revolution in life sciences? Not yet. The ability to seamlessly incorporate knowledge resources for biomedical informatics has been reached neither by bench users nor by application developers. Essential types of life sciences data such as those related to biomarkers, microarrays, mass spectrometry, and many types of fluorescent imaging, are still accessed from domain-specific applications that work with specialized data formats, without any support for semantics and RDF. In order to reach medical and bioinformatics practitioners, RDF must be incorporated transparently, “under the hood”, into the most popular applications, without requiring users to know about the underlying technologies such as OWL, RDF, and SPARQL. The appropriate semantic types for any given study should ideally be semi-automatically derived and assigned during the course of data collection and analysis in order to avoid uncertainty about the semantics later. The incorporation of explicit semantics into applications and even data collection requires a “long view” toward the potential that all artefacts of research, from data to software, should eventually be reusable. Even without a Semantic Web that spans the globe, the benefits of semantic disclosure can be enjoyed within the enterprise and the department, where the cost of reusing code and sharing data is lowered to the point that it actually becomes viable. However, the benefits of a Semantic Web approach have yet to reach many managers and application developers in bioinformatics, most of whom continue to encode implicit semantics into application logic and databases. Perhaps, the Semantic Web in life sciences is just beyond its hype, and it is in the phase where new effective solutions are starting to emerge: while “Semantic Web” is less frequently cited in biomedical publications, statistics which can be related to its use, such as the number of tools and their downloads, are increasing.

### The Killer application

It probably will not be a single “killer application” that brings us to the “inflection point” that tips data toward a full incorporation of semantics and RDF - there are too many application domains and data types for any single application to bring bioinformatics or biomedical research over the threshold.

To trace a comparison, an XML revolution has already quietly taken place and most applications now export and import data in some XML format, without the need for special parsers - XML did not require a “killer app”. A similarly quiet Semantic Web revolution will reach life scientists at the bench, likely after it has been used to integrate across departmental boundaries in the health care clinics, where there seems to be more incentive and means for integration of patient data. In order to reach the life sciences, the RDF representation for each data and instrumentation type must be created and export functionality incorporated into the most important applications. For example, when microarray data can be loaded in RDF directly from ArrayExpress or GEO into an R library that then automatically saves the lists of differentially expressed genes along with their p-values into a triple store (and automatically upload the same explicit information to ArrayExpress and GEO to be associated with a publication), without requiring the data analyst or bioinformatician to know anything about OWL or SPARQL, then the groundwork will have been laid for (automatically) linking microarray study results to other types of biological knowledge.

### The role of pharma

With the pharmaceutical industry hitting the “Intellectual Property cliff” for many drugs (i.e. where they can no longer collect fees for the use of therapeutic compounds they have developed), there seems to arise a corresponding shift toward data sharing. In a keynote address to the Life Sciences Momentum conference in the Netherlands [37], David Cox of Pfizer convincingly described the strategic importance of partnering with data stewards in academia in the search for genetic variants in biobanks, with transparency and agreement to publish results to the public domain. Also, the Pistoia Alliance [38], with many big pharmaceutical members, aims to pool pre-competitive information resources among pharmaceutical companies. The trend continues in the form of the Innovative Medicine Initiative (IMI) [39], European grants with pharma matching sponsorship. Many of the funded projects are looking at how they can share data. In order to share data, organizations must agree on the vocabularies and semantics that they will use to annotate or “package” their resources for sharing. Welcome to the Semantic Web. Not as a visionary and revolutionary tool this time, but as a more mature Semantic Web, with more resources and a clearer focus for its applications.

## A work in progress

In this special issue of the Journal of Biomedical Semantics we present selected papers from the SWAT4LS workshop held in Amsterdam on November 20th, 2009. We see in these papers how the Semantic Web is increasingly being adopted to support the definition of biomedical ontologies, to map between the OBO and OWL ontology languages [31], or to provide a Logic-bases assessment of the compatibility of UMLS ontology sources [40]. We see how it may support the organization of information resources and processes, as in the semantic-based composition of EMBOSS services [41] or in mining semantic networks of bioinformatics e-resources from the literature [42]. Finally, we see how the Semantic Web is reaching new areas in life sciences, as a result of linking the resource description framework to chemioinformatics and proteochemometrics [43].

The diffusion of the Semantic Web in life sciences is a work in progress and is bounded by how easily it can be understood and used by a large number of users. The Semantic Web is inherently simple, but some concepts and terminologies can be confusing, especially because many developers in life sciences are familiar with the object-oriented paradigm and XML. Without a proper introduction, it is easy to misunderstand the role and relations between XML, RDF and OWL. Also, without an introduction to the proper technology, a task such as data integration can become more challenging than necessary. Thus, the adoption of the Semantic Web in the life sciences is effectively bound by the availability of specialized courses, tutorials, and introductory books which can easily reach bioinformaticians and researchers in the life sciences, and which can convey a key message: the Semantic Web is simple.

## Competing interests

The authors declare that they have not competing interests.
